# Clinical Experience of Use of Kampo Medicine Shakuyakukanzoto for Muscle Spasms Caused by Tetanus

**DOI:** 10.7759/cureus.40612

**Published:** 2023-06-18

**Authors:** Kiyohiro Oshima, Yusuke Sawada, Yuta Isshiki, Yumi Ichikawa, Kazunori Fukushima, Yuto Aramaki

**Affiliations:** 1 Department of Emergency Medicine, Gunma University Graduate School of Medicine, Maebashi, JPN

**Keywords:** sedatives, emergency critical care, muscle spasm, kampo medicine (japanese herbal medicine), tetanus

## Abstract

Background

Tetanus is an infectious disease caused by *Clostridium tetani*, which produces tetanospasmin. Intensive care using sedatives and muscle relaxants is required for the management of severe tetanus, however, long-term use of those medicines is associated with the occurrence of post-intensive care syndrome (PICS). Shakuyakukanzoto (SKT), which is clinically used for the treatment of pain associated with sudden myospasm widely, is one of Kampo medicines, and some studies showed that they are effective in treating muscle spasms caused by tetanus. The purpose of this study is to evaluate the usefulness of SKT in the management of tetanus patients from the viewpoint of the reduction of sedatives.

Methods

Patients who were diagnosed with tetanus and were treated in our hospital between January 2006 and December 2022 were included. Patients were divided into two groups, patients treated with SKT and those without SKT, and the background information and clinical courses, especially the reduction of sedatives, were compared between the two groups.

Results

There were five tetanus patients who were treated with SKT (SKT(+) group) and two tetanus patients without SKT (SKT(-) group), respectively. Intubation and mechanical ventilation were required for the management of generalized seizures in all seven patients, who were admitted to the intensive care unit (ICU). The administration of propofol could be discontinued after an average of 8.6 days (range: 3-22 days) from the initiation of SKT administration. The dosage of propofol was lower in patients who received SKT versus those who did not; midazolam and fentanyl exhibited a similar tendency. The mean durations of ICU and hospital stays for patients treated with or without SKT were almost equal (the mean durations of ICU stay in SKT(+) and SKT(-) groups were 22.6 and 24.0 days, and those of hospital stay in SKT(+) and SKT(-) groups were 35.2 and 36.0 days, respectively). All seven patients were discharged and transferred to another hospital for rehabilitation.

Conclusions

SKT may be useful in the management of myospasms in patients with tetanus. It may also prevent the occurrence of PICU in patients with tetanus who require intensive care by reducing the use of sedatives and analgesics.

## Introduction

Tetanus is an infectious disease caused by *Clostridium tetani*, which produces tetanospasmin. Estimating the true burden of tetanus is difficult because most cases occur in low-income and middle-income countries where surveillance systems are limited [1]. Kyu et al. [2] reported that 79% of deaths due to tetanus (44,612 out of 56,743) in 2015 were estimated to occur in South Asia and sub-Saharan Africa. However, the most accurate epidemiological data available are on neonatal tetanus incidence and tetanus that occurs outside the neonatal period is not a notifiable disease in many countries and few countries where tetanus is common have robust reporting systems or accurate incidence data [1]. On the other hand, cases of tetanus in high-income countries are reported on occasion [3,4]. In the UK between 2010 and 2014, two to seven cases were annually reported [5]. In France, 70 patients with tetanus (median age of 80 years) were admitted to intensive care units (ICU) between 2000 and 2014, and 10 (14%) of these patients died [1]. A higher incidence has been reported in Japan, where a national database survey reported 499 tetanus cases between 2010 and 2016, probably due to low immunity in older individuals [6].

Muscle spasm is a characteristic clinical symptom of tetanus, and general management is complicated by the occurrence of generalized seizures. Muscle relaxation, sedation, and the prevention of thromboembolic, respiratory, and other complications comprise the principles of the treatment of tetanus patients especially in the ICU [7]. However, evidence to guide optimal management is scarce because most cases of tetanus occur in resource-limited settings where doing clinical trials is challenging [1].

Benzodiazepines such as diazepam and midazolam, which often in very high doses, have been the mainstay of controlling muscle spasms [8]. In addition, severe spasms might necessitate the use of neuromuscular blocking agents, and the use of such drugs requires the use of mechanical ventilation. The usefulness of propofol [9] and dexmedetomidine [10] for the management of severe tetanus has been reported in recent years. However, heavy sedation, immobility, extended duration of mechanical ventilation, and prolonged ICU stay accompanying long-term use of those medicine described above are associated with the occurrence of post-intensive care syndrome (PICS) [11].

Shakuyakukanzoto (SKT) is one of Kampo medicines, and is clinically used for the treatment of pain associated with sudden myospasm widely. Recently, some studies showed that they are effective in treating muscle spasms caused by tetanus [12-15]. Especially, Nakae et al. reported that the use of SKT reduced the dosage of propofol which was administered for the control of spastic convulsion in a tetanus patient [12]. The purpose of this study is to evaluate the usefulness of SKT in the management of tetanus patients from the viewpoint of the reduction of sedatives.

## Materials and methods

This retrospective study was approved by the research ethics board of Gunma University Hospital (Maebashi, Japan) without the need for informed consent (#2018-130). The conduct of this study was announced on the website of our university.

Patients who were diagnosed with tetanus and were treated in our hospital between January 2006 and December 2022 were included. The diagnosis of tetanus was reached based on symptoms and the clinical course. Patients were divided into two groups, patients treated with SKT and those without SKT. The background information and clinical courses, especially the reduction of sedatives, were compared between the two groups based on data collected from the medical records.

Statistical analysis was not performed in this study because the number of patients was too small to perform statistical analysis.

## Results

Nine patients with tetanus were treated in our hospital between January 2006 and December 2022. SKT was not administered to four of those patients for the following reasons. Of those four patients, two patients were treated before the publication of the first report which revealed the efficacy of SKT in this setting [12], and those two patients were included in this study. However, residual two patients who were not treated with SKT were excluded because one did not have generalized spasms and the other had cardiac arrest prior to treatment initiated at our hospital and passed away within 48 hours of hospitalization.

There were five tetanus patients who were treated with SKT (SKT(+) group) and two tetanus patients without SKT (SKT(-) group), respectively. Hence, SKT was administered to five patients at a daily dose of 7.5 g (2.5 g/pack × 3).

The background information and clinical courses of patients who received SKT (n=5) and those who did not (n=2) are shown in Table 1. Due to the small sample size, it was not possible to perform statistical analysis.

**Table 1:** Background information and clinical courses of patients. DM: diabetes mellitus; HT: hypertension; CI: cerebral infarction; Af: atrial fibrillation; Fent: fentanyl; Mdz: midazolam; Prop: propofol; SKT: shakuyakukanzoto

	Case	Age, years	Sex	Comorbidities	Tetanus vaccine at injury	Total duration of administration, days			Total dosage, mg		
						Prop	Mdz	Fent	Prop	Mdz	Fent
SKT (+)	1	70	Male	DM, HT, Post-CI	-	30	18	7	86,814	3,817	6.8
	2	56	Female	-	-	5	-	13	4,156	-	16.9
	3	63	Female	HT	+	23	19	25	60,857	2,418	46.8
	4	76	Male	HT, Af	-	9	1	9	14,488	26.5	8.0
	5	84	Female	HT	-	4	-	16	1,788	-	12.9
Mean		69.8				14.2	12.7	14	33,621	2,087	18.3
SKT (−)	6	83	Female	-	-	17	14	-	22,545	2,504	-
	7	66	Female	-	-	15	18	18	42,070	4,622	27.0
Mean		74.5				16	16	18	32,308	3,563	27.0

The mean age and male/female ratio of patients treated with SKT were 69.8 years (range: 56-84 years) and 2:3, respectively. Patients who received SKT were younger than those who did not. All seven patients were injured prior to the occurrence of tetanus. Although they consulted physicians immediately after injury, only one patient received a tetanus vaccine (Table 1). All seven patients diagnosed with tetanus were transferred to our hospital for the management of general conditions. Intubation and mechanical ventilation were required for the management of generalized seizures in all seven patients, who were admitted to the ICU. Continuous intravenous administration of a muscle relaxant was performed in only one patient who did not receive SKT (Case 7). Table 1 shows that the total duration of propofol, midazolam, and fentanyl administration tended to be shorter in patients treated with SKT versus those not treated with this agent. The total dosages of midazolam and fentanyl were lower in patients who received SKT versus those who did not; nonetheless, the dosage of propofol was almost equal between the two groups.

The time of SKT initiation and duration of administration are shown in Table 2.

**Table 2:** Clinical courses of patients. The total duration of administration (days) and total dosages of Prop, Mdz, and Fent were identical to those shown in Table 1 (Cases 6 and 7). * Terminated prior to SKT administration. Fent: fentanyl; Mdz: midazolam; Prop: propofol; SKT: shakuyakukanzoto

	Case	Initiation of SKT (hospital days)	Total duration of SKT administration	Total duration of administration and total dosage after SKT administration			Duration of mechanical ventilation (days)	Duration of ICU stay (days)	Duration of hospital stay (days)
				Prop, days (mg)	Mdz, days (mg)	Fent, days (mg)			
SKT (+)	1	30	16	6 (11,220)	-*	-*	37	31	45
	2	3	32	3 (1,716)	-	3 (2.7)	13	20	34
	3	2	48	22 (60,708)	19 (2,418)	24 (46.8)	36	39	51
	4	6	15	8 (14,061)	1 (27)	8 (7.7)	13	15	20
	5	1	21	4 (1,788)	-	16 (12.9)	14	16	26
Mean		8.4	26.4	8.6 (17,899)	10 (1,222)	13 (17.4)	22.6	24.2	35.2
SKT (−)	6	-	-	18 (22,545)	14 (2,504)	-	17	20	28
	7	-	-	15 (42,070)	18 (4,622)	17 (27.0)	31	23	44
Mean				16.5 (32,308)	16 (3,563)	17 (27.0)	24.0	21.5	36.0

The administration of SKT was initiated later in Case 1 compared with the other four cases; this is because this case was treated only six months after the first report which demonstrated the efficacy of SKT against tetanus [12]. The administration of propofol could be discontinued after an average of 8.6 days (range: 3-22 days) from the initiation of SKT administration. The administered dosage of propofol tended to be lower in patients who received SKT versus those who did not; midazolam and fentanyl exhibited a similar tendency (Table 2). The mean durations of ICU and hospital stays for patients treated with or without SKT were almost equal. Finally, all seven patients were discharged from our hospital and transferred to another hospital for rehabilitation.

Below, we present the clinical course of Case 5.

Case 5, a female in her 80s diagnosed with tetanus, was transferred to our hospital. She sustained an injury on her left leg from a cultivator on a farm nine days before admission to our hospital. She was admitted to the ICU and intubated; mechanical ventilation was also initiated. Propofol was concurrently administered for sedation and spasmolysis. SKT was administered on the first hospital day, while treatment with propofol was discontinued on the fourth hospital day. The duration of mechanical ventilation, ICU stay, and hospital stay were 14, 16, and 26 days, respectively. Thereafter, the patient was transferred to another hospital for rehabilitation.

## Discussion

Intensive interventions, spasm control, sedation, mechanical ventilation, and management of complications are generally required for the treatment of tetanus [16]. The administration of a muscle relaxant through continuous infusion is useful in the management of tetanus [7]. The first report on the continuous administration of propofol in this setting was published in 1988 [7]. Subsequently, Sebel et al. reported that propofol provided sedation, a decrease in sympathetic activity, muscle relaxation, and spasm control [17]. PICS is defined as a new or worsening impairment in the physical, cognitive, or mental health status arising and persisting after hospitalization for critical illness [18]. Recently, PICS received considerable research attention. Heavy sedation, immobility, extended duration of mechanical ventilation, and prolonged ICU stay are associated with the occurrence of PICS [11]. Therefore, the excessive use of muscle relaxants and sedatives, such as propofol, may increase the risk of developing PICS.

The Kampo medicine SKT is composed of two types of crude drugs, namely shakuyaku (main component: paeoniflorin) and kanzo (main component: glycyrrhizin). It is generally used for the control of pain associated with sudden myospasms. SKT relaxes skeletal muscles by blocking neuromuscular synapses [19], without affecting normal physiological twitching [20,21]; this is one of the mechanisms involved in peripheral muscle relaxation. Paeoniflorin enhances non-contractile Ca2+ mobilization, which may induce desensitization of the nicotinic acetylcholine receptor at the neuromuscular junction [22]. Moreover, glycyrrhizin depresses contractile Ca2+ mobilization. The inhibition of twitch response through combination treatment with paeoniflorin and glycyrrhizin is pharmacologically attributed to the complementary effects of these compounds on intracellular Ca2+ mobilization [19,22].

In the present cases, the duration of administration and dosage of propofol were shortened and reduced, respectively, after the initiation of treatment with SKT. In addition, midazolam and fentanyl exhibited a similar tendency. Imai et al. reported that glycyrrhizin inhibits the production of prostaglandins and exerts an analgesic effect [23]. Nakae et al. suggested that SKT relieves muscle ischemia caused by muscle contractions, as well as the consequent myalgia [14]. Therefore, SKT may prevent the occurrence of PICS in patients with tetanus who require intensive care by reducing the use of sedatives (e.g., propofol and midazolam) and analgesics (e.g., fentanyl).

Pseudohyperaldosteronism is a major side effect of kanzo [13]. Thus, when treating patients with SKT, physicians should pay attention to the potential occurrence of hypokalemia caused by pseudohyperaldosteronism. Fortunately, in the present cases, hypokalemia was not detected in any of the patients during the clinical course.

Tetanus is a vaccine-preventable disease, and prophylaxis countermeasures (e.g., thorough wound management and vaccination) play the most important role in preventing the occurrence of tetanus. This disease remains prevalent in numerous low- and middle-income countries [1]. Kyu et al. estimated that 79% of deaths due to tetanus (44,612/56,743) occurred in South Asia and sub-Saharan Africa [2]. Considering its cost-effectiveness (i.e., 6.9 Japanese yen/g; 1 US dollar = 140.22 Japanese yen in June 2023), SKT may be a suitable therapeutic agent in those countries.

Limitations

This study, which was performed only in one institute, included a very small sample size. The statistical analysis could not be performed because of the very small sample size. Most cases of tetanus occur in resource-limited settings where doing clinical trials is challenging [1], and there are not many occurrences of tetanus in developed countries including Japan. The National Institute of Infectious Diseases in Japan reported that the number of tetanus patients in Japan is about 100 per year [24]. In addition, the clinical department which takes charge of tetanus patients is different in each hospital. Therefore, case collection reaches extremely difficult. Nevertheless, we believe that the possibility of the usefulness of SKT for the management of tetanus patients could be shown through this study. Of course, further studies are required in the future for the establishment of the usefulness of SKT for the management of tetanus patients.

## Conclusions

The total administered duration and total dosage of sedatives and analgesics were shortened and reduced, respectively, after the initiation of treatment with SKT. SKT may be a useful treatment option for myospasms in patients with tetanus. In addition, SKT may have a possibility to prevent the occurrence of PICS in patients with tetanus who require intensive care.
